# A Single Vaccination of Chimeric Bivalent Virus-Like Particle Vaccine Confers Protection Against H9N2 and H3N2 Avian Influenza in Commercial Broilers and Allows a Strategy of Differentiating Infected from Vaccinated Animals

**DOI:** 10.3389/fimmu.2022.902515

**Published:** 2022-07-08

**Authors:** Yi-xue Sun, Zheng-rong Li, Peng-ju Zhang, Jin-hong Han, Hai-yang Di, Jia-yi Qin, Yan-long Cong

**Affiliations:** ^1^ Laboratory of Infectious Diseases, College of Veterinary Medicine; Key Laboratory of Zoonosis Research, Ministry of Education, Jilin University, Changchun, China; ^2^ Jilin Research and Development Center of Biomedical Engineering, Changchun University, Changchun, China; ^3^ Institute of Animal Biotechnology, Jilin Academy of Agricultural Sciences, Changchun, China; ^4^ Department of Disease Prevention and Control, Zoological and Botanical Garden of Changchun, Changchun, China

**Keywords:** influenza virus, H9N2 and H3N2 subtypes, VLP, chimeric vaccine, DIVA

## Abstract

H9N2 and H3N2 are the two most important subtypes of low pathogenic avian influenza viruses (LPAIV) because of their ongoing threat to the global poultry industry and public health. Although commercially available inactivated H9N2 vaccines are widely used in the affected countries, endemic H9N2 avian influenza remains uncontrolled. In addition, there is no available avian H3N2 vaccine. Influenza virus-like particles (VLPs) are one of the most promising vaccine alternatives to traditional egg-based vaccines. In this study, to increase the immunogenic content of VLPs to reduce production costs, we developed chimeric bivalent VLPs (cbVLPs) co-displaying hemagglutinin (HA) and neuraminidase (NA) of H9N2 and H3N2 viruses with the Gag protein of bovine immunodeficiency virus (BIV) as the inner core using the Bac-to-Bac baculovirus expression system. The results showed that a single immunization of chickens with 40μg/0.3mL cbVLPs elicited an effective immune response and provided complete protection against H9N2 and H3N2 viruses. More importantly, cbVLPs with accompanying serological assays can successfully accomplish the strategy of differentiating infected animals from vaccinated animals (DIVA), making virus surveillance easier. Therefore, this cbVLP vaccine candidate would be a promising alternative to conventional vaccines, showing great potential for commercial development.

## Introduction

Avian influenza is a highly infectious viral disease of birds caused by influenza A virus (IAV) of the family *Orthomyxoviridae* (1). Since the first outbreak in Italy in 1878, avian influenza has spread widely around the world, not only seriously affecting the poultry industry and national food supply and safety (2), but also posing a serious threat to public health (3). Based on the antigenic differences of the two membrane glycoproteins hemagglutinin (HA) and neuraminidase (NA), avian influenza viruses are classified into 16 HA subtypes and 9 NA subtypes (1). According to the pathogenicity to chickens, avian influenza strains are classified as low pathogenic avian influenza viruses (LPAIVs) and highly pathogenic avian influenza viruses (HPAIVs) (4). Although HPAIVs have been paid extensive attention, LPAIV cannot be ignored. Epidemiological investigations have shown that H9N2 is one of the most widespread subtypes of the AIV family, which has been found in wild birds as an enzootic pathogen in poultry across much of Asia and parts of Africa since 1966 (5, 6). Although H9N2 usually shows mild symptoms when infecting poultry, the significant reduction in production performance caused by co-infection of H9N2 with other pathogens is second only to HPAIV (7). Notably, H9N2 AIVs have evolved to break through the interspecies barrier for transmission to humans since 1998 (8) and are been deemed as an emerging challenge (9). As the second most important LPAIV, H3N2 in wild birds and poultry has frequently come into our attention in recent years (10–18). Of particular concern is that human H3N2 pandemic and seasonal influenza may be associated with the prevalence of H3N2 AIVs (19–22), thus highlighting the importance of H3N2 AIVs in public health. Altogether, these remind us that we should pay attention not only to the veterinary significance of H9N2 and H3N2 LPAIVs, but also to their zoonotic potential.

Vaccination is the most effective and cost-effective means of preventing AIV infection. Large-scale vaccination with commercially available monovalent or mixed H9N2 inactivated vaccines contributes substantially to the control of H9N2 outbreaks (6). However, endemic H9N2 avian influenza, especially those caused by the G57 genotype of the virus, has not been effectively controlled (23–26). In addition, there is no available avian H3N2 vaccine. Currently, almost all commercial AI vaccines are inactivated, with usage rate as high as 95.5% (27). However, the traditional inactivated vaccines also have some limitations, although they play a key role in preventing AIV infection. For example, the production of such vaccines relies heavily on specific pathogen-free (SPF) chicken embryos, which would generate a large amount of biohazardous products. In addition, inactivated vaccines cannot implement strategies to differentiate infected from vaccinated animals (DIVA), which makes virus surveillance difficult (28, 29). Of particular note is a recent finding that inactivated vaccine does not prevent H9N2 virus transmission in chickens (30). All of these underscore the necessity to use new technologies to develop vaccines so as to meet the requirements of modern poultry industry for the prevention of epidemic diseases.

Virus-like particles (VLPs) are highly structured, hollow protein particles assembled by one or more structural proteins of a virus. VLPs are highly safe because they have no viral nucleic acid and therefore do not have the ability to replicate independently, nor do they have the potential for genetic recombination or reassortment and virulence recovery. Similar to the morphology of natural viral particles, VLPs can be presented to immune cells in a form close to the true viral conformation. This makes them readily recognized by the immune system, which thus effectively induces humoral and cellular immune responses. Moreover, VLPs can induce effective cytotoxic immune responses (31). In addition, VLPs can be prepared in a variety of expression systems with relatively short preparation cycles, and are easily genetically modified to produce more neutralizing antibodies (28). In particular, VLPs contain only immunogenic proteins and do not induce antibodies to viral internal proteins, which allow DIVA to be serologically tested against the viral proteins that are not incorporated into VLPs. Thus, non-infectious VLPs have emerged as one of the most promising vaccine alternatives to traditional inactivated vaccines.

Considering the dual threat of H9N2 and H3N2 subtypes of LPAIVs to poultry and public health, the development of a bivalent influenza vaccine is essential. Previous studies have showed that the AIV matrix 1 (M1) protein can accommodate a variety of viral surface proteins, demonstrating its potential to incorporate foreign proteins as a universal core protein of VLP (32–35). Therefore, it is feasible to design a multi-subtype VLP approach to simultaneously elicit specific immunity to multiple influenza subtypes, with no requirement for mixing individual vaccines. To display more antigenic proteins on the surface of VLPs, in this study, we replaced the M1 protein of AIV with the Gag protein of bovine immunodeficiency virus (BIV) to construct chimeric bivalent VLPs (cbVLPs) that co-express the major surface antigens of H9N2 and H3N2 AIVs. This bivalent vaccine candidate has not only shown good immunogenicity and protective efficacy against H9N2 and H3N2 AIV challenges in commercial broiler chickens, but also makes the DIVA strategy feasible.

## Materials and Methods

### Cells and Viruses

*Spodoptera frugiperda* (Sf9) insect cells (ATCC, CRL-1711) were maintained in SF900III insect serum-free medium (SFM) (Gibco, USA) supplemented with 5% fetal bovine serum (Gibco, USA) at 27°C. Sf9 suspension cells were cultured in suspension in Sf-900™ II insect serum free suspension medium (Gibco, USA) at 27°C in spinner flasks at a speed of 120rpm. Chicken embryo fibroblast (CEF) cells (ATCC, CRL-11268) were cultured in Dulbecco’s modified Eagle medium (DMEM) (Gibco, USA) supplemented with 10% fetal bovine serum (FBS) (Gibco, USA) and antibiotics at 37°C with 5% CO_2_. AIV strains A/chicken/Jilin/DH109/2012 (H9N2) (abbreviated as DH109) and A/chicken/Guangxi/165C7/2014 (H3N2) (abbreviated as 165C7) were propagated in 9-11-day-old SPF chicken embryonated eggs (Beijing Merial Vital Laboratory Animal Technologies Co., LTD, Beijing, China) and used for further experiment.

### Preparation of Antisera

The anti-AIV polyclonal sera were prepared as described previously (36). Briefly, 3-week-old SPF chickens (Beijing Merial Vital Laboratory Animal Technologies Co., LTD, Beijing, China) were immunized intramuscularly with 0.5mL of 0.1% paraformaldehyde-inactivated DH109 or 165C7 (64 HAU) in the presence of Montanide adjuvant. On 21 days post-immunization (dpi), the collected sera were inactivated at 56°C for 50min and pretreated with receptor-destroying enzyme (neuraminidase from *Vibrio cholerae*) (Sigma-Aldrich, USA) overnight at 37°C to remove nonspecific inhibitors. The pooled sera were identified by hemagglutination inhibition (HI) assay (37) and kept at −20°C until use.

### Generation of a Series of Constructs to Package VLPs

**Figure 1:**
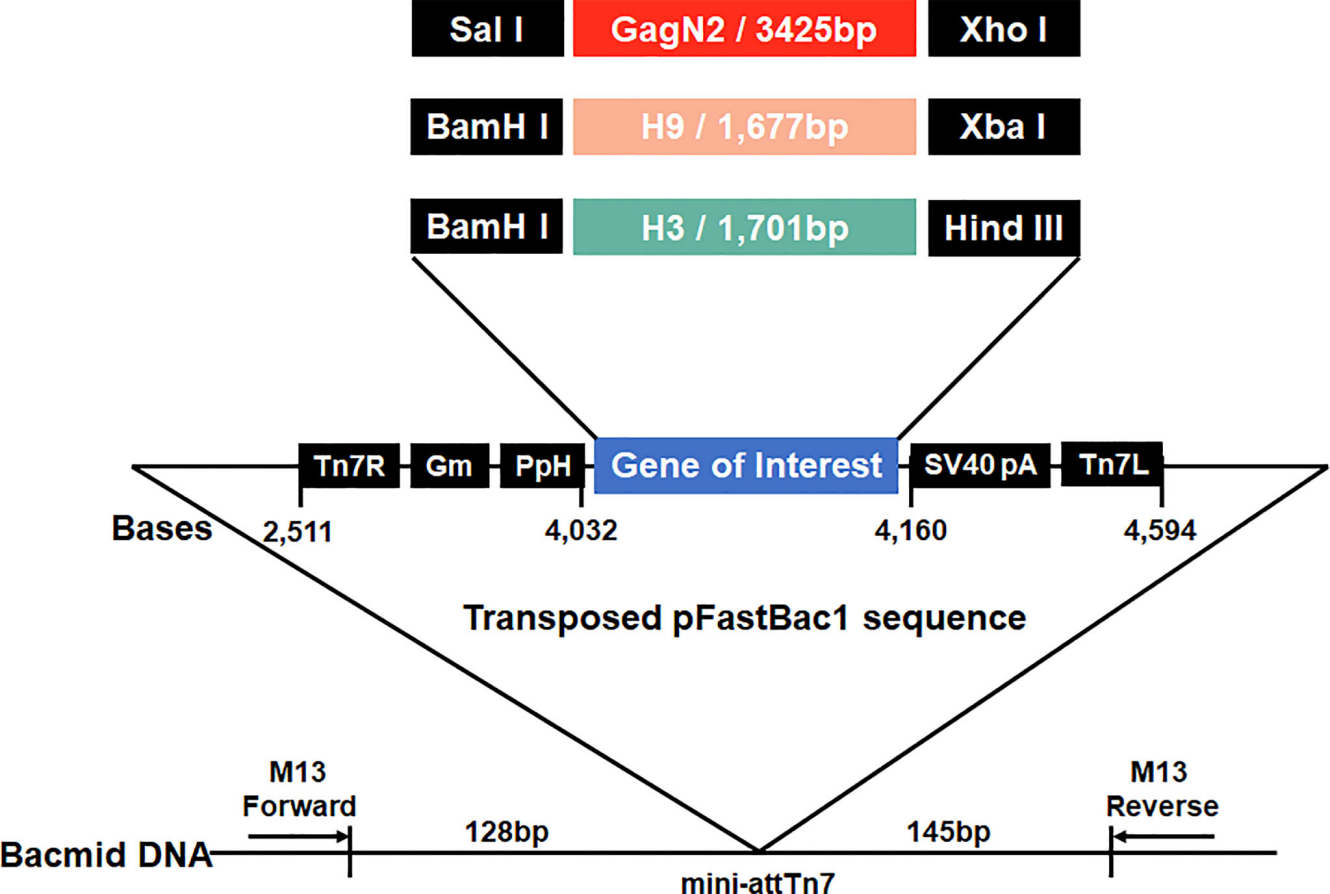
Schematic construction map of three recombinant bacmids. Three expression cassettes harboring the GagN2, H9 or H3 genes limited by Tn7R and Tn7L in the pFastBac1 plasmids will be transposed into the bacmids, respectively. Black arrows show M13 forward and reverse primers used for the PCR analysis of three recombinant bacmids. The diagram is not to scale.

To generate constructs to package VLPs, the full-length H9 and H3 HA nucleotide sequences derived from DH109 (GenBank accession no. KF886409) and 165C7 (KT022317) were amplified by RT-PCR using the primers as shown in **Table 1**. The gene sequence encoding the Gag (L04974) of BIV linked with the N2 (KF886411) of DH109 by an internal ribosome entry site (IRES) sequence was synthesized biochemically (Genscript, Nanjing, China). The synthesized gene was named GagN2. Then, these three genes were introduced into the pFastBac1 transfer vector (Thermo Fisher Scientific, USA) to construct the pFastBac1-GagN2, pFastBac1-H9 and pFastBac1-H3. The recombinant baculoviruses (rBV) expressing GagN2, H9 and H3 genes were generated using the Bac-to-Bac baculovirus expression system. In brief, the three correctly sequenced recombinant shuttle plasmids were each transformed into *E. coli* competent cells *DH10Bac* (Invitrogen, USA) for homologous recombination. After inoculating an appropriate amount of bacterial fluid on the selected solid LB medium containing 100µg/mL X-gal, 10µg/mL tetracycline, 40µg/mL IPTG, 7µg/mL gentamicin and 50µg/mL kanamycin at 37°C for 24h, the white single colonies were selected. The recombinant bacmid (rBacmid) colonies were identified by PCR with the universal M13 primers (forward primer: 5′-GTTTTCCCAGTCACGAC-3′, reverse primer: 5′-CAGGAAACAGCTATGAC-3′) to obtain positive rBacmid-GagN2, rBacmid-H9 and rBacmid-H3 (**Figure 1**). The three rBacmids were transfected into Sf9 insect cells using TransIT^®^-293 (Mirus, USA). The genome of the 3^rd^ generation of recombinant baculovirus (rBV) was extracted and identified by PCR with M13 primers. Meanwhile, rBV-GagN2, rBV-H9 and rBV-H3 were identified by hemagglutination assay (38) and Western blot (39), and their stock titers were determined by the BacPAK™ baculovirus rapid titer kit (Clontech Labs, USA).

**Table 1:** The information of primers used to amplify the GagN2, H9, and H3 genes in order to construct the recombinant pFastBac1 plasmids.

Genes	Primer names	Sequences (5′→3′)^*^	Restriction enzymes
GagN2	Gag-F	AAGTCGACATGAAGAGAA	Sal I
	N2-R	CCTCGAGTTATATAGGCAT	Xho I
H9	H9-F H9-R	CGGATCCATGAGTCTTCTGACC GCTCTAGATCACTTGAACCGCTG	BamH I Xba I
H3	H3-F	CGGATCCATGAAGACAACCATT	BamH I
	H3-R	CCAAGCTTCTATCAGTTTACTCAAATG	Hind III

^*^The sequence recognized by each restriction enzyme is underlined.

### Assembly and Identification of VLPs

**Figure 2:**
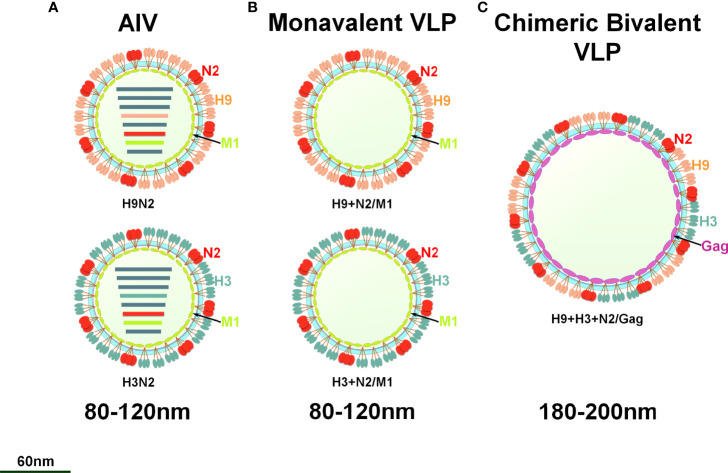
A schematic diagram of monovalent VLPs and bivalent chimeric bivalent VLP. **(A)** A sketch illustrates H9N2 or H3N2 subtypes of AIV; **(B)** Monovalent H9N2 or H3N2 VLPs with HA, NA and M1 genes; **(C)** Chimeric bivalent VLPs co-localizing H9, H3 and N2 into the Gag of BIV.

To prepare the VLP vaccine, 5 multiplicity of infection (MOI) of rBV-GagN2, rBV-H9 and rBV-H3 were co-infected into 2.5×10^6^/mL of Sf9 cells for 96h at 27°C, and then the cell suspension was collected. The assembled chimeric bivalent VLPs (cbVLPs) were concentrated and purified by 20%-40%-60% sucrose gradient centrifugation and characterized by hemagglutination assay (38), virus elution assay (40), Western blot (39) and transmission electron microscope (TEM) (41). The schematic diagram of the constructed cbVLPs is shown in **Figure 2**.

### Preparation of cbVLP Immunogen

The purified cbVLPs were diluted with PBS solution. Then, Imject™ alum adjuvant (Thermo, USA) and diluted cbVLPs were completely formulated at a ratio of 1:1 (w/w) on a magnetic stirrer and stored at 4°C until use. The content of cbVLPs was quantified at 40μg/0.3mL, 30μg/0.3mL and 15μg/0.3mL for the three dose groups according to the instructions of Pierce™ BCA protein assay kit (Thermo, USA). Inactivated H3N2 vaccine was prepared by formulating 0.1% paraformaldehyde-inactivated H3N2 virus with Imject™ alum adjuvant to obtain final concentrations of 40μg/0.3mL.

### Vaccination and Challenge

Ten-day-old commercial white-finned broilers (Jilin Deda Co., Ltd., Jilin, China) with maternal antibodies below 2log_2_ were randomly divided into 6 groups of 30 chickens each. Referring to the instructions for the commercial inactivated vaccine based on A/duck/Nanjing/01/1999 (H9N2) (Shandong Huahong Biological Engineering Co., LTD, Binzhou, Shandong, China), chickens were intramuscularly immunized with 40μg, 30μg and 15μg of cbVLPs, commercial H9N2 vaccine and inactivated H3N2 vaccine, respectively. Chickens in all groups were immunized with 0.3mL each according to the recommended immunization dose of the commercial H9N2 vaccine. Three weeks after the single immunization, chickens in each group were randomized into two subgroups, one for intranasal virus challenge with 10^6^EID_50_/100μL of H9N2 or H3N2 virus strains and the other for continued monitoring of changes in antibody titers.

### Lymphocyte Proliferation Test

The lymphocyte proliferation test is a classic test to detect cellular immune function, which reflect the activity and functional status of lymphocytes in response to relevant stimuli (42). To investigate the effect of cbVLPs constructed in this study on the immune function of splenic lymphocytes, we examined the proliferation of splenic lymphocytes from immunized chickens using Cell Counting Kit-8 (CCK-8) (GLPBIO, USA). Briefly, 1×10^6^/100μL of lymphocyte suspension was added to a 96-well plate and incubated at 37°C for 2h with concanavalin A (ConA, Sigma) with the final concentration of 50μg/mL, or 20μL of inactivated AIV antigen (1 × 10^5^TCID_50_/mL) in each well, respectively. The plate was incubated at 37°C for 20h, and then added methylthiazoltetrazolium (MTT) (5mg/mL) with 10μL/well for further incubation at 37°C for 4h. Finally, the reaction was stopped with 100μL of 10% dimethyl sulfoxide (DMSO, Solarbio) in each well. The optical density (OD) value at 490nm was detected with a microplate reader, and then the stimulation index (SI) of splenic lymphocyte was calculated with the following formula: SI = (OD sample well − OD blank well)/(OD negative well − OD blank well).

### Hemagglutination Inhibition and Virus Neutralizing Assays

After immunization, blood was collected weekly and the antibody titers in serum were measured by HI and virus neutralizing (VN) assays as described (37, 43), where DH109 and 165C7 were used as viral antigens of H9N2 and H3N2, respectively. Briefly, for the HI assay, chicken serum was serially diluted 2-fold and incubated with 4 HA units of viral antigen for 30 min at 37°C, followed by incubation with 25μL of 1% chicken red blood cells. Plates were read after 30min of incubation at room temperature (RT). The homologous HI titers were expressed as the reciprocal of the highest serum dilution that completely inhibited hemagglutination and converted into log_2_ values. For the VN assay, sera were serially diluted with DMEM containing 1% FBS and mixed with equal volume of 100TCID_50_ of H9N2 or H3N2 virus strains. After incubation at 37°C for 1h, the virus-serum mixture was inoculated onto a monolayer of CEF cells in a 96-well plate and incubated for 4 days. The VN antibody titer was determined as the reciprocal of the highest serum dilution that completely inhibited the cytopathic effect (CPE) caused by viral infection.

### Bodyweight Monitoring

Clinical signs of chickens in each group were observed twice daily after the challenge with DH109 or 165C7 and the bodyweight of each chicken was monitored before feeding on 3, 5, 7, 10, and 14 days post-challenge (dpc). The bodyweight before challenge was served as 100% and the relative change of bodyweight after challenge was calculated.

### qRT-PCR

To monitor virus shedding, oropharyngeal and cloacal swabs were collected every other day since 3dpc from 8 randomly selected chickens in each group. Samples were sterilized overnight in PBS containing 1% penicillin and streptomycin and centrifuged at 2,000g for 10min. 200μL of sample solution was inoculated into the allantoic cavity of 10-day-old SPF embryonated eggs. Three days later, viral RNA was extracted from the allantoic fluids using the QIAamp Viral RNA Mini Kit (Qiagen, USA) and the matrix (M) gene of AIV were identified by one-step quantitative RT-PCR (qRT-PCR) with the specific M primers (forward primer: 5′-CTTCTAACCGAGGTCGAAACG-3′, reverse primer: 5′-GGCATTTTGGACAAAGCGTCTA-3′). The cycle threshold values of the strain to be tested were evaluated by the constructed standard curve.

### Immunohistochemical Assay

To determine virus replication in chickens, paraffin sections of the trachea and lungs were made and the distribution of the virus was observed by immunohistochemical (IHC) assay as described (44). Briefly, tissues from IAV-infected chickens were fixed in phosphate-buffered 10% formalin solution, then paraffin-embedded, sectioned, and incubated for 1h at RT for IAV staining using a nucleoprotein (NP)-specific monoclonal antibody to IAV (prepared by our laboratory). Detection was performed with a secondary biotinylated goat anti-mouse IgG antibody (Chemicon, USA) at 1:1,0000 for 1h at RT, streptavidin–horseradish peroxidase (Roche, USA) at 1:5,000 for 1h at RT, and OPD substrate (Sigma, USA), followed by hematoxylin was used as a counterstain before mounting with glycerol gel.

### DIVA

To differentiate serologically between cbVLPs-vaccinated chickens and infected chickens, AIV was detected using a commercial competitive ELISA (cELISA) kit according to the manufacturer’s instructions (BioNote, USA). Briefly, the cELISA plate, which is pre-coated with AIV NP antigen, was incubated with an equal mixture of sample and HRP-conjugated anti-NP monoclonal antibodies for 30 min at 37°C. After incubation, all unbound material was removed by three washes prior to addition of a substrate solution. Sample values were calculated according to the following formula: Sample values=1-(OD_450_ sample/mean OD_450_ negative), and sample value <0.50 was considered negative for the presence of antibodies to NP. Since the cbVLPs developed in this study do not contain the NP protein of AIV, it is expected that the level of anti-NP antibodies to AIV will increase only after viral infection.

### Statistical Analysis

Significance of difference between data was analyzed by a Two-Way ANOVA test in GraphPad Prism 8.0 software (GraphPad Software Inc., San Diego, California USA). *P >* 0.05 means no significant difference (ns) and *P <* 0.05 is considered statistically significant (* for *P <* 0.05, ** for *P <* 0.01, *** for *P <* 0.001, **** for *P <* 0.0001).

## Results

### Recombinant Bacmids Were Identified by PCR

After homologous recombination in *DH10Bac*, the recombinant shuttle plasmids of pFastBac1-GagN2, pFastBac1-H9 and pFastBac1-H3 were screened for resistance for three consecutive times, and the recombinant bacmids were extracted and identified by PCR with M13 primers. The results showed that the amplification products containing the GagN2, H9 and H3 fragments were approximately 5,725bp, 4,001bp and 3,983bp, respectively, which were consistent with the expected sizes (**Figure S1**).

### Recombinant Baculoviruses Were Identified and Quantified

**Figure 3:**
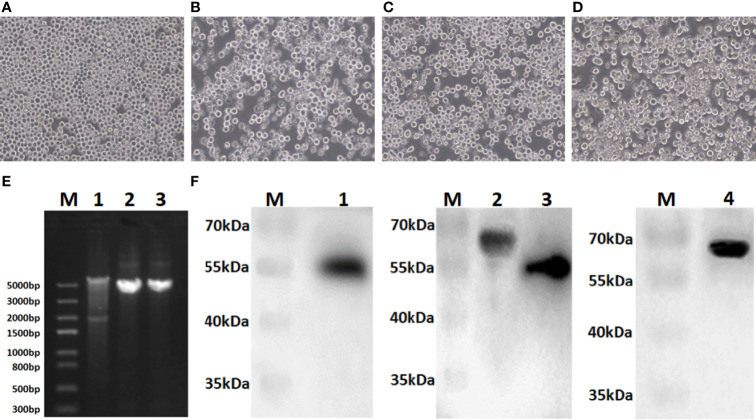
Identification of recombinant baculoviruses. Panel **(A)** Normal Sf9 cells; Panels **(B–D)** indicate the cytopathic effect of Sf9 cells after infection with recombinant baculoviruses rBV-GagN2, rBV-H9 or rBV-H3. Panel **(E)** indicates the PCR identification of recombinant baculovirus genomes with M13 primers. Lane M: Trans5K DNA marker; Lane 1: rBV-GagN2; Lane 2: rBV-H9; Lane 3: rBV-H3. The GagN2, H9 and H3 fragments were approximately 5,725bp, 4,001bp and 3,983bp, respectively. Panel **(F)** indicates the Western blot result of the foreign proteins expressed by recombinant baculoviruses. Lane M: Protein molecular weight standard; Lane 1: Gag; Lane 2: H9 HA; Lane 3: NA; Lane 4: H3 HA. The molecular weights of Gag, H9, N2, and H3 were approximately 54kDa, 63kDa, 52kDa, and 63kDa, respectively.

Sf9 cells were infected with recombinant bacmids rBacmid-GagN2, rBacmid-H9 and rBacmid-H3, respectively. The pathological changes of Sf9 cells were observed at 72h after incubation at 27°C. As shown in **Figures 3A–D**, compared to normal cells, the cells infected with recombinant baculoviruses (rBVs) became larger and rounder, cell proliferation was slow or even stopped, and lysed cell debris was observed in the supernatant. The extracted genomes of the third generation rBVs were identified by PCR using M13 primers, and the fragment sizes were consistent with expectations (**Figure 3E**). Furthermore, the supernatants of cells infected with rBVs were collected to analyze the expression of the target proteins by Western blot using polyclonal antibodies against Gag protein, H9N2 AIV and H3N2 AIV. Additionally, the supernatants of cells infected with rBVs were collected and the expression of target proteins was analyzed by Western blot using polyclonal antibodies against the Gag protein of BIV or against H9N2 AIV and H3N2 AIV. As shown in **Figure 3F**, the protein bands of Gag, H9, N2, and H3 were approximately 54kDa, 63kDa, 52kDa, and 63kDa, respectively, corresponding to the expected sizes. Further, the titers of the fourth generation rBVs were determined by recombinant baculovirus titer assay kit. The results showed that the titers of rBV-GagN2, rBV-H9 and rBV-H3 were 3.67×10^7^PFU/ml, 2.74×10^7^PFU/ml and 2.85×10^7^PFU/ml, respectively.

### The cbVLPs Were Successfully Constructed and Validated by Hemagglutination Assay, Virus Elution Assay, Western Blot and TEM

**Figure 4:**
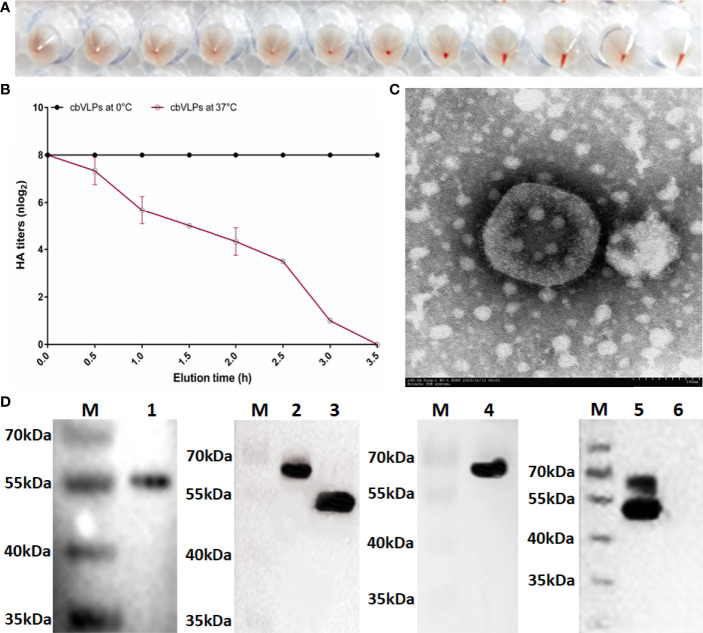
Identification of cbVLPs. Panel **(A)**: The hemagglutination activity of cbVLPs. The HA titer of cbVLPs can reach 8log2. Panel **(B)**: Elution profiles of cbVLPs from the adhered chicken red blood cells. In the virus elution assay, 25μL of cbVLPs with an HA titer of 1:256 was incubated with 25μL of 1% chicken red blood cells in U-shape microtiter plates at 4°C for 1h. Subsequently, the plates were incubated at 37°C. Reduction of HA titers was recorded. Panel **(C)**: A near-spherical enveloped cbVLP (diameter ≈180nm) with spikes. Scale bar 100nm. Panel **(D)**: Identification of the components of cbVLPs by Western blot. Lane M: Protein molecular weight standard; Lane 1: Gag protein detected by Gag antibody; Lane 2: H9 protein detected by H9N2 antibody; Lane 3: N2 protein detected by H9N2 antibody; Lane 4: H3 protein detected by H3N2 antibody; Lane 5: The upper overlapping bands of H9 and H3 and the lower overlapping bands of Gag and N2, which were simultaneously detected by the mixture of antibodies against Gag, H9N2 and H3N2. Line 6: The Sf9 cell suspension as a negative control. The molecular weights of Gag, H9, N2, and H3 were approximately 54kDa, 63kDa, 52kDa, and 63kDa, respectively.

The HA protein of AIV has the ability of agglutinating erythrocytes. If the cbVLP was successfully constructed, it would have agglutinating activity. As shown in **Figure 4A**, the HA titer of cbVLPs can reach 8log_2_. Meanwhile, the virus elution assay showed that N2 has neuraminidase activity that cleaves the binding between HA and its receptor on the surface of erythrocyte (**Figure 4B**), thus demonstrating the presence and function of N2. Under TEM, the morphological structure of cbVLPs resembles that of natural viruses, with a spherical shape, spikes attached to the surface, and a particle size of approximately 180nm, which exceeds the diameter of natural AIV particles (80-120nm) (**Figure 4C**). To further determine whether the cbVLPs were successfully assembled in the Bac-to-Bac baculovirus expression system, the components of cbVLPs were identified by Western blot. As shown in **Figure 4D**, four target bands corresponding to the expected size were observed simultaneously.

### The cbVLPs Can Induce Effective Cellular and Humoral Immune Responses

**Figure 5:**
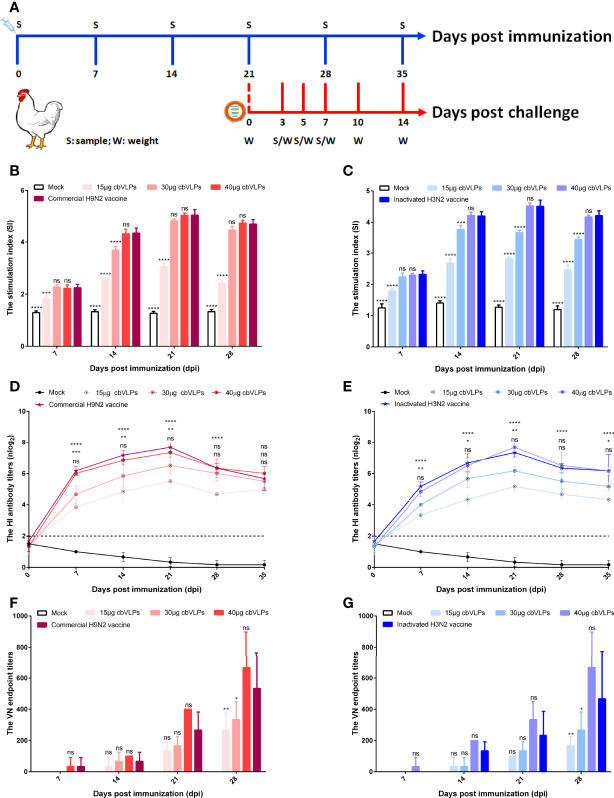
The immune response of chickens after immunization with cbVLPs. Panel **(A)**: Animal experimental design for immunization and challenge. Panels **(B, C)**: The splenic lymphocyte proliferation after stimulation with H9N2 or H3N2 AIV strains, respectively. The results were presented in the form of the stimulation index (SI), which reached a peak on 21dpi. Panels **(D, E)**: The dynamic change of HI antibody titers in the sera against homologous H9N2 or H3N2 AIV strains, respectively. A sustained increase in the HI antibody titers was observed in all cbVLP groups up to 3 weeks after immunization. Panels **(F, G)**: The VN antibody titers against homologous H9N2 or H3N2 AIV strains, respectively. As can be seen the trend is similar to what is observed in Panels **(D)** or **(E)**, but with higher levels of antibodies against corresponding antigen. Significance of difference was compared between cbVLPs and commercial H9N2 vaccine or inactivated H3N2 vaccine by a Two-Way ANOVA test. Statistical differences were indicated by asterisk (ns P > 0.05, *P < 0.05, **P < 0.01, ***P < 0.001, ****P < 0.0001).

To assess the immunogenicity of cbVLPs, ten-day-old commercial white-feather broiler chickens were immunized intramuscularly with 0.3mL of PBS, 15μg cbVLPs, 30μg cbVLPs, 40μg cbVLPs, commercial H9N2 vaccine, and inactivated H3N2 vaccine. On day 21 after a single immunization, each group of chickens was randomly divided into two subgroups, with one subgroup receiving an intranasal challenge with either H9N2 or H3N2 AIV strains and the other for continued monitoring of changes in antibody titers. The schedule on immunization and challenge of chickens is shown in **Figure 5A**. Splenic lymphocytes from chickens on 7, 14, 21 and 28 dpi were isolated for lymphocyte proliferation tests. The results showed that the SI values of each immunization group were higher than that of the mock group and reached a peak on 21dpi. The comparisons of cbVLPs groups with different immunization doses showed that the SI values were dose-dependent. However, there was no significant difference in the SI values at each time point between only the 40μg cbVLPs and the commercial H9N2 vaccine or the inactivated H3N2 vaccine prepared in this study (*P >* 0.05) (**Figures 5B, C**). In addition, a sustained increase in the HI antibody titers against homologous H9N2 or H3N2 AIV strains was observed in all cbVLP groups up to 3 weeks after immunization (**Figures 5D, E**). Furthermore, the HI antibody titers of cbVLP groups were higher than the protective line level (2log_2_), even on 35dpi. Similarly, the HI antibody titers in the three cbVLPs groups were dose-dependent. At each time point, only 40μg cbVLPs induced comparable HI antibody titers to those of commercial H9N2 vaccine or inactivated H3N2 vaccine (*P >* 0.05). A similar profile was observed for the specific VN antibody response against H9N2 or H3N2 AIVs (**Figures 5F, G**). All these results suggest that cbVLPs can induce effective immune response to H9N2 and H3N2.

### A Single Vaccination of cbVLP Confers Protection Against H9N2 and H3N2 Avian Influenza

**Figure 6:**
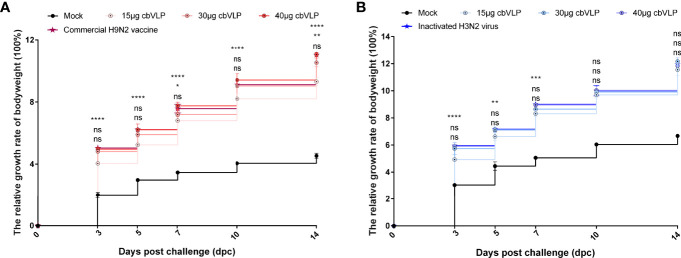
The changes of bodyweights of those immunized chickens after challenge with H9N2 or H3N2 AIV strains. Panel **(A)**: H9N2 challenge; Panel **(B)**: H3N2 challenge. Significance of difference was compared between cbVLPs and commercial H9N2 vaccine or inactivated H3N2 vaccine by a Two-Way ANOVA test. There was no significant difference in growth rate between the 40μg cbVLP group and the vaccine groups. Statistical differences were indicated by asterisk (ns P > 0.05, *P < 0.05, **P < 0.01, ***P < 0.001, ****P < 0.0001).

To investigate the protective efficacy of cbVLPs against H9N2 and H3N2 AIV infections, chickens were intranasally challenged on 21dpi. The results showed that all chickens survived the 14-day observation period after challenge, with only those in the mock group exhibiting mild clinical signs such as mild depression, anorexia, respiratory distress and reduced activity levels, which peaked between 4 and 6dpc. The bodyweight of chickens in each group continued to grow steadily, and there was no significant difference in growth rate between the 40μg cbVLP group and the vaccine groups (*P >* 0.05) (**Figure 6**).

From the onset of 3dpc, the oropharynx and cloaca of each group of chickens were examined for virus shedding by qRT-PCR every other day. The results showed that there was no virus shedding in the 40μg and 30μg cbVLP groups after H9N2 AIV challenge. Although there was existence of virus in the two groups of 15μg cbVLPs and commercial H9N2 vaccine, no virus was detected again on 5 dpc and 7dpc (**Table 2**). After challenge with H3N2 AIV, no viral shedding was detected in the 40μg, 30μg and 15μg cbVLP groups, while virus was detected on 3dpc in the inactivated H3N2 vaccine group, and no virus was excreted on 5dpc and 7dpc in all immunization groups (**Table 2**).

**Table 2:** *In vitro* detection of virus shedding in the oropharyngeal and cloacal swabs from those immunized chickens on 3, 5, and 7 days post challenge (dpc) with H9N2 or H3N2 AIV strains.

Groups	Challenge	Oropharyngeal swabs			Cloacal swabs			Virus shedding/Total
		3dpc	5dpc	7dpc	3dpc	5dpc	7dpc	
40μg cbVLPs	H9N2	0/8	0/8	0/8	0/8	0/8	0/8	0/8
30μg cbVLPs		0/8	0/8	0/8	0/8	0/8	0/8	0/8
15μg cbVLPs		1/8	0/8	0/8	3/8	0/8	0/8	3/8
Commercial H9N2 vaccine		3/8	0/8	0/8	2/8	0/8	0/8	3/8
Mock		8/8	8/8	8/8	8/8	8/8	8/8	8/8
40μg cbVLPs	H3N2	0/8	0/8	0/8	0/8	0/8	0/8	0/8
30μg cbVLPs		0/8	0/8	0/8	0/8	0/8	0/8	0/8
15μg cbVLPs		0/8	0/8	0/8	0/8	0/8	0/8	0/8
Inactivated H3N2 vaccine		1/8	0/8	0/8	2/8	0/8	0/8	2/8
Mock		8/8	8/8	8/8	8/8	8/8	8/8	8/8

**Figure 7:**
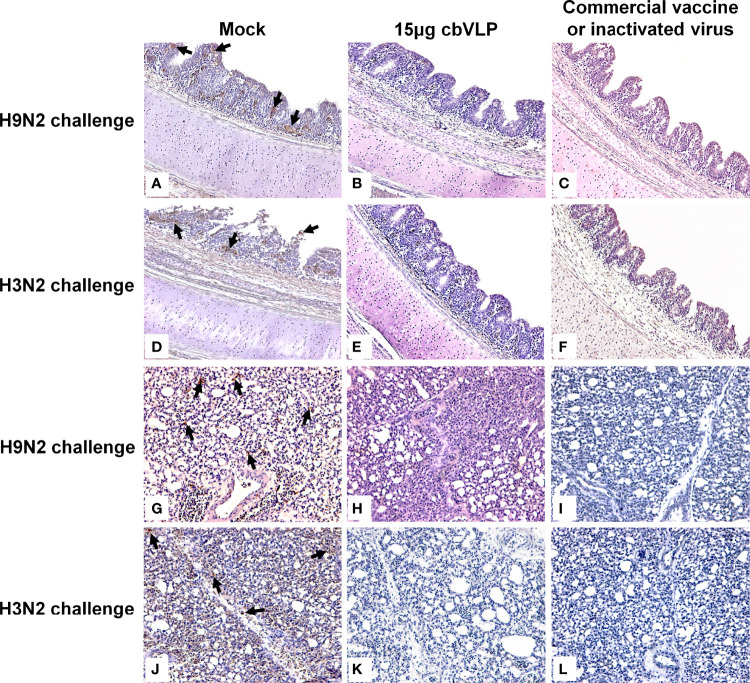
Immunohistochemistry analysis of tissue samples from immunized chickens intranasally inoculated with H9N2 or H3N2 AIV strains on 7 days post challenge. Panels **(A–F)** indicate those trachea and lungs from the groups of mock, 15μg cbVLPs and commercial H9N2 vaccine after H9N2 challenge. Panels **(G–L)** indicate those trachea and lungs from the groups of mock, 15μg cbVLPs, and inactivated H3N2 vaccine after H3N2 challenge. Only the trachea and lungs in the mock groups showed positive antigens for H9N2 or H3N2 AIVs. Magnification: 20×.

To assess the inhibitory effect of cbVLPs on systemic transmission of the virus, tissues and organs from randomly selected chickens were collected every other day after challenge, ground and inoculated into 10-day-old SPF chicken embryonated eggs. The virus in the allantoic fluids was detected by qRT-PCR. The results showed that although the H9N2 and H3N2 AIV strains used in this study had broad tissue tropisms, there was existence of virus in part of tissues and organs on 3dpc only in the groups of 15μg cbVLPs, commercial H9N2 vaccine and inactivated H3N2 vaccine (**Tables 3**, **4**). No virus was detected on 5dpc and 7dpc in all immunization groups. Additionally, the viral load in the trachea and lungs on 7dpc was also detected. The IHC results showed the presence of viral antigens only in the mock groups after challenge with H9N2 or H3N2 AIV strains (**Figure 7**).

**Table 3:** *In vivo* distribution of H9N2 AIV in those immunized chickens on 3, 5, and 7 days post challenge.

Groups	dpc	Brain	Trachea	Lung	Intestines	Pancreas	Spleen	Kidney	Muscle
40μg cbVLPs	3	–	–	–	–	–	–	–	–
30μg cbVLPs		–	–	–	–	–	–	–	–
15μg cbVLPs		–	+	+	+	–	–	–	–
Commercial H9N2 vaccine		–	+	+	+	+	–	–	–
Mock		–	+	+	+	+	–	+	–
40μg cbVLPs	5	–	–	–	–	–	–	–	–
30μg cbVLPs		–	–	–	–	–	–	–	–
15μg cbVLPs		–	–	–	–	–	–	–	–
Commercial H9N2 vaccine		–	–	–	–	–	–	–	–
Mock		–	+	+	+	+	+	+	+
40μg cbVLPs	7	–	–	–	–	–	–	–	–
30μg cbVLPs		–	–	–	–	–	–	–	–
15μg cbVLPs		–	–	–	–	–	–	–	–
Commercial H9N2 vaccine		–	–	–	–	–	–	–	–
Mock		+	+	+	+	+	+	+	+

"+" and "-" indicate that the virus was detected or not detected in the corresponding tissue, respectively.

**Table 4:** *In vivo* distribution of H3N2 AIV in those immunized chickens on 3, 5, and 7 days post challenge.

Groups	dpc	Brain	Trachea	Lung	Intestines	Pancreas	Spleen	Kidney	Muscle
40μg cbVLPs	3	–	–	–	–	–	–	–	–
30μg cbVLPs		–	–	–	–	–	–	–	–
15μg cbVLPs		–	–	–	–	–	–	–	–
Inactivated H3N2 vaccine		–	+	+	+	+	–	–	–
Mock		–	+	+	+	+	+	+	–
40μg cbVLPs	5	–	–	–	–	–	–	–	–
30μg cbVLPs		–	–	–	–	–	–	–	–
15μg cbVLPs		–	–	–	–	–	–	–	–
Inactivated H3N2 vaccine		–	–	–	–	–	–	–	–
Mock		+	+	+	+	+	+	+	+
40μg cbVLPs	7	–	–	–	–	–	–	–	–
30μg cbVLPs		–	–	–	–	–	–	–	–
15μg cbVLPs		–	–	–	–	–	–	–	–
Inactivated H3N2 vaccine		–	–	–	–	–	–	–	–
Mock		+	+	+	+	+	+	+	+

"+" and "-" indicate that the virus was detected or not detected in the corresponding tissue, respectively.

### The cbVLPs Can accomplish a successful DIVA strategy

**Figure 8:**
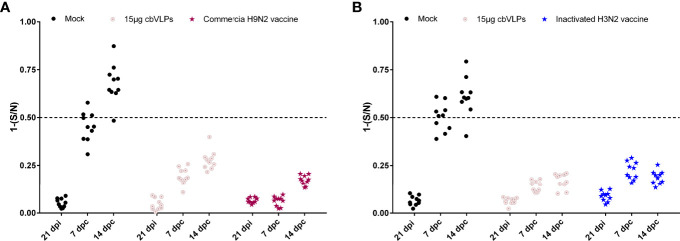
Differentiation of vaccinated chickens from infected chickens. Ten serum samples were collected on 21 days post immunization and on 7 and 14 days post challenge. The levels of serum antibody against the nucleoprotein (NP) of IAVs were tested with a commercially available NP-cELISA kit. Each dot represents the NP-specific antibody value of each chicken. Panel **(A)**: The immunization groups post challenge with H9N2 AIV; Panel **(B)**: The immunization groups post challenge with H3N2 AIV. Significance of difference was compared between cbVLPs and commercial H9N2 vaccine or inactivated H3N2 vaccine by a Two-Way ANOVA test. dpi, day post immunization; dpc, day post challenge.

For the purpose of DIVA, chicken sera were collected on 0dpi and 21dpi and on 7dpc and 14dpc for avian influenza NP-cELISA testing. As shown in **Figure 8**, anti-NP antibodies were negative in the sera sampled from the groups of 15μg cbVLPs, commercial H9N2 vaccine and inactivated H3N2 vaccine before and after the challenge. However, in the mock group, 3 and 9 out of 10 serum samples were positive on days 7 and 14 after H9N2 challenge, while 6 and 9 out of 10 serum samples were positive on days 7 and 14 after H3N2 challenge. This indicates that cbVLPs with companion serological tests can accomplish a successful DIVA strategy.

## Discussion

There are two glycoprotein spikes embedded on the surface of IAV, HA and NA, which are the major protective antigens. Although it is known that the amount and immunogenicity of NA are lower than HA, the role of NA in vaccine protection cannot be ignored. A growing body of literature suggests that NA is a secondary target for vaccine design and that a combination of NA and HA would be more effective in blocking the release of newborn virus and widen the breadth of protection against IAV (45–50). In the case of H9N2 and H3N2 LPAIVs, their HA genes belong to two different phylogenetic groups (51), which means that there is no or incomplete cross-protection between them (52). Nevertheless, the same N2 isoforms of H9N2 and H3N2 IAVs share homologous sequences and similar antigenic structures, resulting in the cross-reactivity (53). For those whole virus inactivated vaccines, such as the commercially available H9N2 vaccines, a limitation is that the antigenic abundance of each protein in the virions determines their immunogenicity and protective efficacy (54). Another one is that inactivated vaccine cannot differentiate infected from vaccinated animals.

Nowadays, VLPs have become the focus of development of safe and effective vaccines for many viruses, which have been proposed as an important alternative to conventional vaccines. This is not only because VLPs are safer than attenuated vaccine, but also because in some cases VLPs exceed the immune response elicited by inactivated vaccines (55). In addition, the Bac-to-Bac baculovirus expression system is widely used for VLP production due to its high expression level, eukaryotic processing characteristics and large-scale production capacity (56). Influenza VLPs usually comprise of three strain-specific genes, HA, NA and M1, in which they assemble into regular, highly repeated patterns resembling viral structures. Unfortunately, there are still challenges to be addressed for VLP vaccines, including optimizing the production process to increase antigen yield, minimizing production costs, and enhancing broad protection against multiple variants and subtypes of IAV. In some studies, M1 was used as the inner core of IAV VLPs (32–35), while in other designs, Gag of retrovirus such as BIV, murine leukemia virus, simian/human immunodeficiency viruses were used in place of M1 (57–60). M1 is a highly conserved capsid protein of IAV that interacts with the cytoplasmic tail regions of HA, NA and M2, directing them toward the plasma membrane to form VLPs (61). However, Gag does not require any assistance to form VLPs as they are self-sufficient and use the host cell protease for the final processing of VLPs. The shape and size of VLPs depend mainly on the capsid proteins and the display proteins on the surface of VLPs (35). When the capsid is served as the inner core of a VLP, the larger its diameter, the larger the diameter of the assembled VLP is usually. It was shown that the average particle diameter of influenza VLPs constructed with Gag of BIV was 150-180nm, which was larger than that of VLPs constructed with M1 at 120-150nm, implying that it would allow the VLPs to accommodate more spikes if Gag with larger surface area was used as a capsid (60). Given the continuing threat of H9N2 and H3N2 AIVs to the poultry industry and public health, we constructed chimeric bivalent VLPs displaying the H9, H3 and N2 proteins of H9N2 and H3N2 AIVs with BIV Gag as the core. The diameter of these cbVLPs is about 180nm under TEM (**Figure 4C**). Based on the estimation that a spherical virion with an average diameter of 120nm has approximately 375 spikes (62), it is speculated that the prepared cbVLPs with a diameter of 180nm could accommodate about 800 spikes, which would increase the content of AIV spike immunogens and thus reduce the production cost of VLPs. More importantly, there is no similarity found in Gag proteins between BIV and avian retrovirus, which greatly facilitates the development of a DIVA vaccine using BIV Gag as a diagnostic marker. Taken together, it is more advantageous to use BIV Gag as the inner core of a VLP vaccine than to use IAV M1. Unfortunately, we did not utilize suitable methods, such as immunoelectron microscopy or immunoprecipitation, to favorably identify H9, H3 and N2 antigens present on the same cbVLP, which is a shortcoming of this study.

To date, a number of monovalent or mixed inactivated H9N2 vaccines are commercially available. The success of vaccination depends to a large extent on the antigenic match between the vaccine strains and the circulating virus strains. To evaluate the immunoprotective efficiency of cbVLPs, whose H9 is derived from DH109, we used a commercial monovalent inactivated H9N2 vaccine prepared with A/duck/Nanjing/01/1999 as a positive control for H9N2. The sequence alignment showed that A/duck/Nanjing/01/1999 and DH109 have 92.65% and 95.71% amino acid identity for HA and NA (**Table S1**), indicating that they have an antigenic gap. The phylogenetic analysis showed that the HA of A/duck/Nanjing/01/1999 belongs to the early BJ94-like subclade, whereas the HA of DH109 clustered into the G57 subclade that has been circulating in recent years (**Figure S2**). It thus implies that A/duck/Nanjing/01/1999 may not match the circulating strains in terms of antigenicity and emphasizes the necessity to develop novel vaccines. In this study, our results showed that 40μg of cbVLPs was sufficient to elicit comparable levels of cellular and humoral responses when compared with a commercial H9N2 vaccine or the inactivated H3N2 vaccine prepared (**Figure 5**). In particular, 40μg of cbVLPs was superior to these two vaccines in inhibiting viral shedding and replication (**Figure 7**). Although we did not identify the immunogenicity of the N2 protein present on the cbVLPs, the virus elution assay confirmed that N2 has neuraminidase activity (**Figure 4B**). This would allow the cbVLPs to “infect” target cells in an infection manner similar to wild virus, thereby facilitating the recognition of cbVLPs by immune cells so as to induce a better immune response. An additional advantage of the cbVLPs produced in this study is that it is able to differentiate between wild virus infection and vaccine immunization, which makes the DIVA strategy feasible (**Figure 8**). It is concluded that a single vaccination of the cbVLPs assembled by embedding H9, H3 and N2 into BIV Gag can confer chickens complete protection against homologous H9N2 and H3N2 AIV infections and make the DIVA strategy feasible, thus showing a good commercial prospect.

## Supplemental Material

### Materials and Methods

#### Phylogenetic Analysis

A phylogenetic tree of HA gene was constructed based on 1,000 bootstrap replicates using the maximum-likelihood method implemented in MEGA 7.0 software, where the best-fit general time-reversible model of nucleotide substitution with gamma-distributed rate variation among sites (with 4 rate categories, Γ4) was used (63).

## Data Availability Statement

The datasets presented in this study can be found in online repositories. The names of the repository/repositories and accession number(s) can be found in the article/**Supplementary Material**.

## Ethics Statement

The animal study was reviewed and approved by Committee on the Ethics of Animal Experiments of Jilin University.

## Author Contributions

Y-lC and P-jZ conceived and designed the experiments. Y-xS, Z-rL, and H-yD participated in the experiments; Y-xS, J-hH and J-yQ analyzed the data. Y-lC, Y-xS, and P-jZ wrote the manuscript. All the authors provided the final approval of the manuscript.

## Funding

This work was supported by the National Natural Science Foundation of China (32072893) and the Plan Project of Jilin Provincial Science and Technology Development (20210101047JC).

## Conflict of Interest

The authors declare that the research was conducted in the absence of any commercial or financial relationships that could be construed as a potential conflict of interest.

## Publisher’s Note

All claims expressed in this article are solely those of the authors and do not necessarily represent those of their affiliated organizations, or those of the publisher, the editors and the reviewers. Any product that may be evaluated in this article, or claim that may be made by its manufacturer, is not guaranteed or endorsed by the publisher.

## Supplementary Material

Supplementary Figure 1 The PCR identification of recombinant bacmids. M: Trans5K DNA marker; 1: rBacmid-GagN2; 2: rBacmid-H9; 3: rBacmid-H3. The GagN2, H9 and H3 fragments were approximately 5,725bp, 4,001bp and 3,983bp, respectively.

Supplementary Figure 2 Phylogenetic tree of the nucleotide sequences of HA genes of H9N2 viruses. The maximum-likelihood tree was constructed by using MEGA version 7.0 (http://www.megasoftware.net/) based on 1,000 replications of bootstrap analysis. Virus subclades are indicated at right. The red solid circle represents the commercial inactivated H9N2 vaccine strain. The blue solid square represents one of two virus strains used to construct the cbVLPs. Scale bar indicates nucleotide substitutions per site.

Supplementary Table 1 Comparison of HA and NA amino acid identity between the commercial H9N2 vaccine strain and those virus strains used to construct the cbVLPs. ^a^Identity comparison of A/duck/Nanjing/01/1999 itself. ^b,c^Identity comparisons between the virus strains used to construct the cbVLPs and A/duck/Nanjing/01/1999.
