# Supplementary figures and images for: A Single Vaccination of Chimeric Bivalent Virus-Like Particle Vaccine Confers Protection Against H9N2 and H3N2 Avian Influenza in Commercial Broilers and Allows a Strategy of Differentiating Infected from Vaccinated Animals
Source: Front Immunol. 2022 Jul 8;13:902515. doi: 10.3389/fimmu.2022.902515 (PMC9304867; doi:10.3389/fimmu.2022.902515)

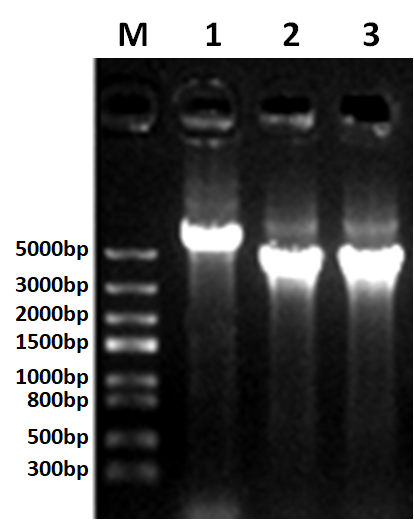

Supplement: Supplementary Figure 1 — The PCR identification of recombinant bacmids. M: Trans5K DNA marker; 1: rBacmid-GagN2; 2: rBacmid-H9; 3: rBacmid-H3. The GagN2, H9 and H3 fragments were approximately 5,725bp, 4,001bp and 3,983bp, respectively. [file Image_1.tif]

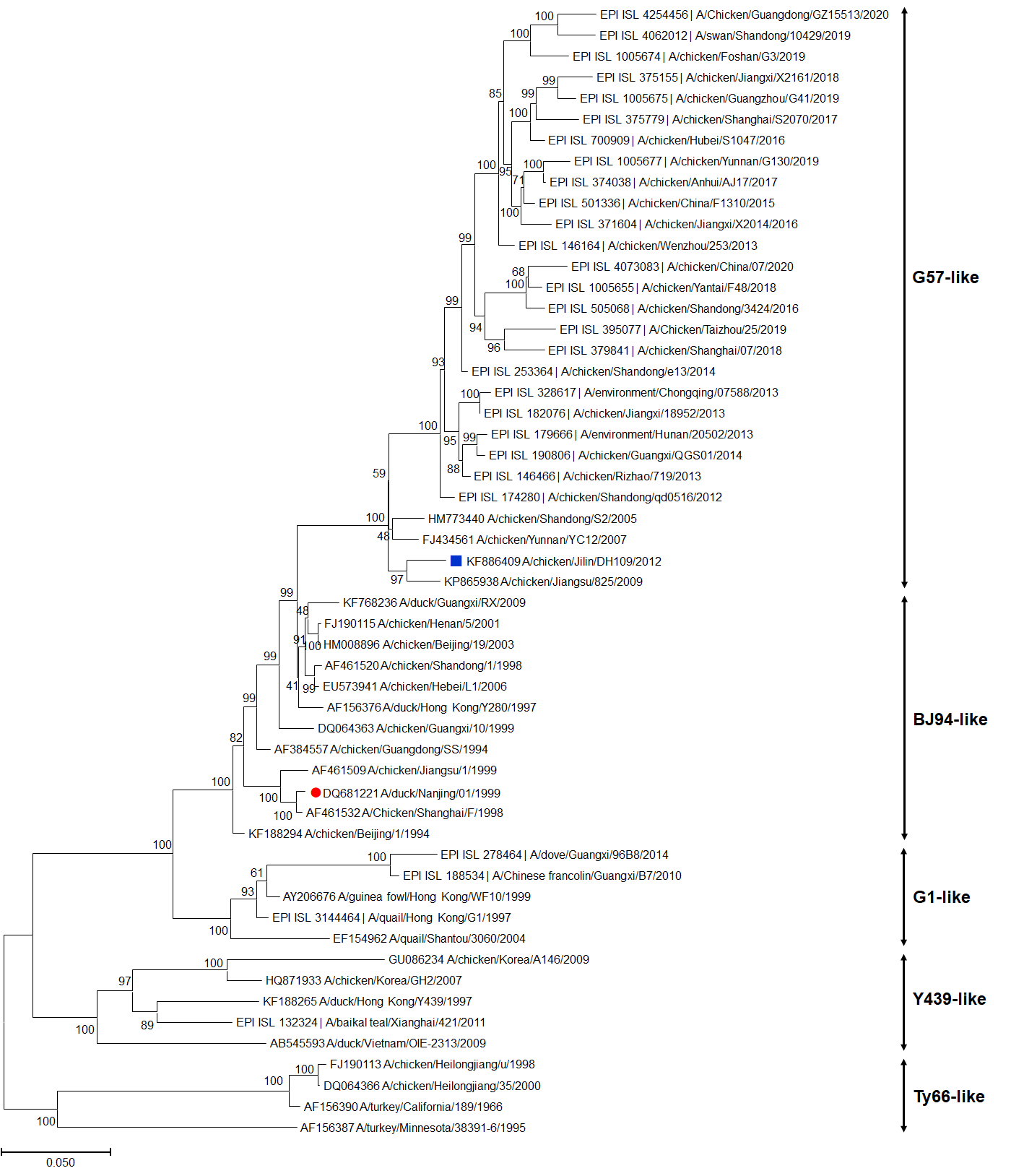

Supplement: Supplementary Figure 2 — Phylogenetic tree of the nucleotide sequences of HA genes of H9N2 viruses. The maximum-likelihood tree was constructed by using MEGA version 7.0 (http://www.megasoftware.net/) based on 1,000 replications of bootstrap analysis. Virus subclades are indicated at right. The red solid circle represents the commercial inactivated H9N2 vaccine strain. The blue solid square represents one of two virus strains used to construct the cbVLPs. Scale bar indicates nucleotide substitutions per site. [file Image_2.tif]
