# Supplementary material for: A Single Vaccination of Chimeric Bivalent Virus-Like Particle Vaccine Confers Protection Against H9N2 and H3N2 Avian Influenza in Commercial Broilers and Allows a Strategy of Differentiating Infected from Vaccinated Animals
Source: Front Immunol. 2022 Jul 8;13:902515. doi: 10.3389/fimmu.2022.902515 (PMC9304867; doi:10.3389/fimmu.2022.902515)
Supplement: Supplementary Table 1 — Comparison of HA and NA amino acid identity between the commercial H9N2 vaccine strain and those virus strains used to construct the cbVLPs. aIdentity comparison of A/duck/Nanjing/01/1999 itself. b,cIdentity comparisons between the virus strains used to construct the cbVLPs and A/duck/Nanjing/01/1999. [file DataSheet_1.docx]

**APPENDIX**

**MATERIALS and METHODS**

**Phylogenetic Analysis**

A phylogenetic tree of HA gene was constructed based on 1,000 bootstrap replicates using the maximum-likelihood method implemented in MEGA 7.0 software, where the best-fit general time-reversible model of nucleotide substitution with gamma-distributed rate variation among sites (with 4 rate categories, Γ4) was used (63).

**REFERENCE**

Kumar, S., G. Stecher and K. Tamura (2016). "MEGA7: Molecular Evolutionary Genetics Analysis Version 7.0 for Bigger Datasets." Mol Biol Evol **33**(7): 1870-1874.

# FIGURE S1 | The PCR identification of recombinant bacmids. M: Trans5K DNA marker; 1: rBacmid-GagN2; 2: rBacmid-H9; 3: rBacmid-H3. The GagN2, H9 and H3 fragments were approximately 5,725bp, 4,001bp and 3,983bp, respectively.

# FIGURE S2 | Phylogenetic tree of the nucleotide sequences of HA genes of H9N2 viruses. The maximum-likelihood tree was constructed by using MEGA version 7.0 (http://www.megasoftware.net/) based on 1,000 replications of bootstrap analysis. Virus subclades are indicated at right. The red solid circle represents the commercial inactivated H9N2 vaccine strain. The blue solid square represents one of two virus strains used to construct the cbVLPs. Scale bar indicates nucleotide substitutions per site.

# TABLE S1 | Comparison of HA and NA amino acid identity between the commercial H9N2 vaccine strain and those virus strains used to construct the cbVLPs.

| Viruses | | Amino acid identity （%） | | GenBank accession no. | |
| --- | --- | --- | --- | --- | --- |
|  |  | HA | NA | HA | NA |
| The commercial inactivated H9N2 vaccine strain | A/duck/Nanjing/01/1999 (H9N2) | 100.00^a^ | 100.00^a^ | DQ681221 | DQ681205 |
| The virus strains used to construct the cbVLPs | A/chicken/Jilin/DH109/2012 (H9N2) | 92.65^b^ | 95.71^b^ | KF886409 | KF886411 |
|  | A/chicken/Guangxi/165C7/2014 (H3N2) | 41.36^c^ | 88.27^c^ | KT022317 | KT022318 |

^a^Identity comparison of A/duck/Nanjing/01/1999 itself.

^b,c^Identity comparisons between the virus strains used to construct the cbVLPs and A/duck/Nanjing/01/1999.
